# Reduced creatinine clearance is associated with early development of subcutaneous tophi in people with gout

**DOI:** 10.1186/1471-2474-14-363

**Published:** 2013-12-21

**Authors:** Nicola Dalbeth, Meaghan E House, Anne Horne, William J Taylor

**Affiliations:** 1 Bone and Joint Research Group, Department of Medicine, Faculty of Medical and Health Sciences, University of Auckland, 85 Park Rd, Grafton, New Zealand; 2 Department of Medicine, University of Otago Wellington, PO Box 7343, Wellington, 6242, New Zealand

**Keywords:** Gout, Tophus, Kidney, Creatinine

## Abstract

**Background:**

Although typically a late feature of gout, tophi may present early in the course of disease. The aim of this study was to identify factors associated with the presence of early tophaceous disease.

**Methods:**

People with gout for <10 years were prospectively recruited, and had a comprehensive clinical assessment including examination for subcutaneous tophi. The clinical factors independently associated with the presence and number of tophi were analyzed using regression models.

**Results:**

Of the 290 participants, there were 47 (16.2%) with clinically apparent tophi. In univariate analysis, those with tophi were older, were more frequently taking diuretics and colchicine prophylaxis, and had lower creatinine clearance. The association between the presence of tophi and creatinine clearance was strongest in those with creatinine clearance ≤30 ml/min. In logistic regression analysis, creatinine clearance ≤30 ml/min was associated with the presence of tophi, even after adjusting for ethnicity, corticosteroid use, colchicine use and diuretic use (multivariate adjusted odds ratio 7.0, p = 0.005). Participants with tophi reported higher frequency of gout flares, pain scores, patient global assessment scores, and HAQ scores.

**Conclusions:**

The presence of tophi is associated with more symptomatic disease in people with gout for <10 years. Creatinine clearance is independently associated with early presentation of subcutaneous tophi.

## Background

The tophus is the pathognomic feature of chronic gout, and represents a chronic foreign-body granulomatous response to monosodium urate (MSU) crystal deposits [1]. In untreated gout, development of subcutaneous tophi is typically a late feature of disease, occurring more than 10 years after development of gout flares [2,3]. Tophi are more frequently observed in people with prolonged disease duration, advanced age, diuretic use, corticosteroid use and solid organ transplantation [3-6]. Gouty tophi have major clinical relevance, as they contribute to musculoskeletal disability and reduced health-related quality of life [7,8]. These lesions are also implicated in joint damage in gout, and are associated with increased risk of mortality in people with gout [9,10].

Although these lesions are typically a late feature of gout, gouty tophi occasionally present early in the course of disease, either as the initial manifestation of gout or within a few years of first gout flare. The factors associated with early presentation of subcutaneous tophi in people with gout have not been reported. The aim of this analysis was to identify factors associated with the presence of early tophaceous disease.

## Methods

Participants were prospectively recruited by community advertising and through primary and secondary care clinics in Auckland and Wellington, New Zealand. Key inclusion criteria were: classification of gout as defined by Wallace [11], and first attack of gout and/or tophus within the last 10 years. The New Zealand Multiregional Ethics Committee approved the study and participants provided written informed consent.

At a study visit, the following data were recorded: demographic data (age, gender, ethnicity), gout history (confirmation of diagnosis, disease duration, frequency of gout flares, days off work due to gout in the preceding three months, gout treatments), medical history and concomitant medications including diuretics, examination (tender (68) and swollen (66) joint counts and subcutaneous tophus count), questionnaires (a 9-item self report adherence questionnaire based on the Medication Adherence Report Scale [12] to assess adherence to urate lowering therapy [13], Health Assessment Questionnaire (HAQ)-II [14], patient global assessment of gout severity visual analogue scale (100 mm) and pain visual analogue scale (100 mm)), laboratory tests (serum urate, creatinine and C-reactive protein). Creatinine clearance was calculated using the Cockcroft-Gault equation [15]. Estimated glomerular filtration rate (eGFR) was determined using the Modification of Diet in Renal Disease formula [16].

The presence and number of subcutaneous tophi were recorded by one of two clinical research assistants with extensive experience in the assessment and measurement of tophi. Flare frequency was self-reported as the number of gout flares in the preceding three months. Disease duration was reported by the patient and was defined as the time from the first clinical manifestation of gout (either flare or tophus).

Data were analysed using SPSS (SPSS Inc., Chicago, IL). Means with standard deviations (SD) and percentages were used to describe the clinical characteristics of participants. Differences between participants with and without tophi were analyzed using chi squared analysis and t tests. Spearman’s correlations were used to determine the relationship between tophus count and other clinical variables. Logistic regression was used to determine the independent clinical variables associated with the presence of tophi. Poisson regression was used to determine the independent clinical variables associated with the subcutaneous tophus count. Clinical factors with p < 0.15 in the univariate analysis for presence of tophi were included in the regression models (in addition to ethnicity). The key variables of interest were pre-specified as the presence of tophi and the number of tophi. Creatinine clearance was pre-specified as the measure of renal function in this analysis. All tests were two tailed and P < 0.05 was considered statistically significant.

## Results

We recruited 290 people with gout for <10 years into the study. Of these, there were 47 (16.2%) participants with clinically apparent tophi. In participants with tophaceous disease, the mean (SD) number of tophi was 3.1 (3.4), and the mean (SD) disease duration was 5.8 (3.2) years. The clinical features of those with and without tophi are shown in Table 1. In univariate analysis, those with tophi were older, were more frequently taking diuretics and colchicine, and had lower creatinine clearance.

**Table 1:** Clinical features of participants with and without tophi

**Variable**	**No tophi (n = 243)**	**Tophi (n = 47)**	**p**
Age, years	57.8 (14.5)	64.1 (14.0)	0.006
Male sex, n (%)	170 (70.0%)	33 (70%)	1.0
Ethnicity			
Caucasian/other, n (%)	182 (74.8%)	36 (77%)	1.0
Maori or pacific, n (%)	61 (25.2%)	11 (23%)	1.0
Disease duration, years	5.1 (3.8)	5.8 (3.2)	0.22
Cardiovascular events, n (%)	76 (31.3%)	20 (43%)	0.18
Type 2 diabetes, n (%)	29 (12%)	8 (17%)	0.34
Hypertension, n (%)	98 (40.3%)	18 (38%)	0.87
Kidney stones, n (%)	17 (7.0%)	5 (11%)	0.37
Diuretic use, n (%)	61 (25.1%)	22 (47%)	0.004
Colchicine use	70 (28.8%)	21 (45%)	0.039
Non-steroidal anti-inflammatory drug use	50 (20.5%)	8 (17%)	0.79
Allopurinol use, n (%)	147 (60.5%)	26 (55%)	0.52
Allopurinol dose, mg/day	215 (92)	213 (116)	0.93
Corticosteroid use, n (%)	29 (11.9%)	10 (21%)	0.10
Low dose aspirin use, n (%)	62 (25.5%)	15 (32%)	0.37
Adherence score*	40.9 (6.2)	40.9 (5.9)	1.0
Body mass index, kg/m^2^	31.3 (6.9)	30.0 (6.5)	0.23
Systolic blood pressure, mmHg	138 (22)	139 (23)	0.68
Creatinine clearance, mL/min	72.1 (28.6)	56.3 (27.4)	0.001
C-reactive protein, mg/L	5.3 (11.9)	8.0 (15.0)	0.17
Serum urate, mmol/L	0.40 (0.13)	0.43 (0.11)	0.25
Gout flare frequency, per 3 months	1.8 (5.9)	5.4 (18.2)	0.01
Tophus count	0 (0)	3.1 (3.4)	<0.001
Tender joint count (/68)	1.6 (3.9)	5.8 (8.7)	<0.001
Swollen joint count (/66)	0.6 (1.8)	3.3 (7.1)	<0.001
HAQ-II	0.43 (0.55)	0.77 (0.73)	0.001
Patient global assessment, mm	21.9 (23.1)	33.7 (26.4)	0.002
Pain visual analogue scale, mm	15.9 (24.2)	24.9 (25.9)	0.021

*for those on urate-lowering therapy.

Unless specified otherwise, data are presented as mean (SD).

Physical examination showed more swollen and tender joints in those with tophi (Table 1). Participants with tophi reported higher frequency of gout flares, pain scores, patient global assessment scores, and HAQ scores. After adjusting for sex, age and ethnicity, the relationship persisted between the presence of tophi and HAQ (p = 0.002), patient global assessment (p = 0.002), and pain visual analogue scale (p = 0.02).

In the entire group of participants, the number of tophi correlated with age, creatinine clearance, but not with body mass index or serum urate concentration (Table 2). The number of tophi also correlated with swollen and tender joint counts, patient global assessment, pain visual analogue, and HAQ scores. Gout flare frequency and C-reactive protein did not correlate with the number of tophi.

**Table 2:** Correlations between tophus count and clinical features in all participants (n = 290)

**Variable**	**R**	**p**
Age	0.15	0.01
Disease duration	0.10	0.09
Body mass index	−0.10	0.10
Systolic blood pressure	0.01	0.83
Creatinine clearance	−0.22	<0.001
C-reactive protein	0.10	0.11
Serum urate	0.08	0.19
Gout flare frequency	0.06	0.33
Tender joint count	0.26	<0.001
Swollen joint count	0.23	<0.001
HAQ-II	0.18	0.003
Patient global assessment	0.20	0.001
Pain visual analogue scale	0.16	0.006

The association between the presence of tophi and creatinine clearance was strongest in those with creatinine clearance ≤30 ml/min (Table 3). In logistic regression analysis, creatinine clearance ≤30 ml/min was associated with the presence of tophi, even after adjusting for ethnicity, corticosteroid use, colchicine use and diuretic use (multivariate adjusted odds ratio 7.0, p = 0.005). After adjusting for creatinine clearance, age and diuretic use were not associated with the presence of tophi (adjusted p = 0.64 and 0.10 respectively). The relationship between renal function and the presence of tophi was confirmed using eGFR as an additional measure of kidney function. In this analysis, eGFR of 30–59 ml/min/1.73 m^2^ was associated with the presence of tophi (unadjusted OR (95% CI) 3.2 (1.3-7.7), p = 0.009), with a similar trend for eGFR < 30 ml/min/1.73 m^2^ (OR (95% CI) 3.8 (0.94-15.1), p = 0.06).

**Table 3:** Relationship between creatinine clearance bands and the presence of tophi: logistic regression analysis

**Creatinine clearance (ml/min)**	**No tophi (n = 243), n (%)**	**Tophi (n = 47), n (%)**	**Unadjusted odds ratio (95% CI)**	**Unadjusted P**	Multivariate* adjusted odds ratio (95% CI)	Multivariate* adjusted P
>90	63 (25.9%)	4 (9%)	1.0 (ref)	-	1.0 (ref)	-
>60-90	90 (37.0%)	14 (30%)	2.4 (0.8-7.8)	0.13	2.2 (0.7-7.3)	0.19
>30-60	74 (30.5%)	19 (40%)	4.1 (1.3-12.7)	0.015	3.0 (0.9-10.0)	0.07
0-30	16 (6.6%)	10 (21%)	9.7 (2.7-35.0)	0.001	7.0 (1.8-27.6)	0.005

*adjusted for ethnicity, corticosteroid use, diuretic use and colchicine use.

In Poisson regression analysis, there was a strong relationship between creatinine clearance bands with the tophus count, even after adjusting for ethnicity, corticosteroid use, colchicine use and diuretic use (Table 4). When analyzing those with at least one tophus (n = 47), creatinine clearance ≤30 ml/min was associated with tophus count (unadjusted relative risk estimate 2.3 (1.02-5.1), p = 0.044). Using eGFR as an additional measure of kidney function, eGFR bands of 30–59 and <30 ml/min/1.73 m^2^ were associated with the number of tophi (unadjusted relative risk estimate (95% CI) 7.2 (4.0-12.9) and 5.4 (2.4-12.1) respectively, p < 0.001 for both).

**Table 4:** Relationship between creatinine clearance levels and the number of tophi: Poisson regression analysis

**Creatinine clearance (ml/min)**	**Unadjusted relative risk estimate (95% CI)**	**Unadjusted P**	Multivariate* adjusted relative risk estimate (95% CI)	Multivariate* adjusted P
>90	1.0 (ref)	-	1.0 (ref)	-
>60-90	2.6 (1.1-5.9)	0.025	2.5 (1.1-5.8)	0.031
>30-60	7.2 (3.3-15.7)	<0.001	6.1 (2.8-13.7)	<0.001
0-30	14.7 (6.6-32.9)	<0.001	12.0 (5.2-27.7)	<0.001

*adjusted for ethnicity, corticosteroid use, diuretic use and colchicine use.

## Discussion

In this study, just over 15% of participants with gout for less than 10 years had tophaceous disease. Creatinine clearance was the major factor independently associated with early presentation with tophi. Although other factors such as diuretic use and older age were associated with presence of tophi in univariate analysis, these factors were not independently associated with early tophaceous disease when creatinine clearance was included in the regression models. These data raise the possibility that kidney disease may play a role in the development of tophaceous disease, or conversely that kidney deposition of MSU crystals causing renal impairment is more common in patients with tophaceous disease.

It is of interest that variables such as ethnicity, sex and body mass index were not associated with early presentation with tophi. In addition, this study did not show a relationship between presence of tophi and disease duration, serum urate concentration, use of urate-lowering therapy, dose of urate-lowering therapy or compliance to urate-lowering therapy. Using the current study design, we cannot exclude the possibility that the duration of hyperuricaemia prior to development of gout or treatment prior to study entry may have contributed to the risk of developing tophi. Although these patients had relatively early disease, the cross-sectional nature of this study does not allow us to determine the direction of the relationship between tophaceous disease and renal impairment; that is, whether patients who were inadequately treated developed tophi and gout-related kidney disease, or whether kidney disease at presentation contributed to development of tophi, even in the presence of adequate gout treatment. It seems likely that the presence of kidney disease would lead to lower excretion of uric acid, and potentially greater urate pool, which may not be entirely reflected by serum urate concentrations [17]. It is also conceivable that chronic kidney disease could promote development of tophi through mechanisms in addition to increased urate pool. Potential mechanisms might include acceleration of MSU crystal nucleation or growth, or altered immune responses to MSU crystals [18].

Our data provide further evidence that the presence of gouty tophi is associated with important functional consequences for people with gout. We have observed that participants with tophi have more evidence of joint inflammation, greater pain scores, and more activity limitation, despite higher use of colchicine prophylaxis. These findings are consistent with other studies of people with gout [7,8], and emphasize the need to carefully examine for tophi even in early disease, and treat intensively in the context of tophaceous disease [19,20]. A concerning observation of this study was the low use of urate-lowering therapy and poor control of serum urate, particularly in those with tophi, which is a recognized indication for pharmacological urate-lowering therapy [19]. These findings are consistent with many other studies of gout management worldwide [21,22]. Our findings support the importance of further patient and practitioner education about the need to examine for tophi (even in patients with early disease), and initiate effective urate-lowering therapy to prevent tophi and dissolve these lesions once they occur.

We acknowledge the potential limitations of this study. Tophi were not microscopically proven, and it is possible that some designated tophi represented other lesions. This analysis has focused on subcutaneous tophi, without the use of advanced imaging methods such as ultrasound or dual energy computed tomography, which may detect many more tophi than are clinically apparent [23]. Furthermore, assessment of joint inflammation, particularly the causes of joint swelling, can be difficult in people with gout, where tophi, osteoarthritis, and synovitis may co-exist [24]. These issues are consistent with the reality of usual clinical practice. In this study, disease duration was defined as the time from the first clinical manifestation of gout (either flare or tophus). Estimation of disease duration may be difficult in gout, especially since tophi may be occur before the onset of flares and may be undetected by both the individual and healthcare professionals. However, the definition used is consistent with virtually all studies of gout, and is likely to be reliable, noting the typically severe symptoms of pain and disability associated with an acute gout flare.

## Conclusions

Tophaceous disease occurs early in the course of gout in approximately 15% people. The presence of tophi is strongly associated with features of chronic joint inflammation, pain and activity limitation. Creatinine clearance is independently associated with the presence of subcutaneous tophi in people with recent onset gout. Prospective longitudinal studies are needed to address the relative importance of chronic kidney disease, urate burden and other risk factors in the development of tophi in people with gout.

## Abbreviations

CI: Confidence interval; eGFR: Estimated glomerular filtration rate; HAQ: Health Assessment Questionnaire; MSU: Monosodium urate; SD: Standard deviation.

## Competing interests

The authors declare that they have no competing interests.

## Author’s contributions

ND (the guarantor) accepts full responsibility for the work and the conduct of the study, had access to the data, and controlled the decision to publish. ND conceived the study, participated in data analysis, and drafted the manuscript, MEH and AH recruited the patients, collected data, and assisted with the analysis, WJT assisted in study design, data analysis, and drafting the manuscript. All authors read and approved the final manuscript.

## Pre-publication history

The pre-publication history for this paper can be accessed here:

http://www.biomedcentral.com/1471-2474/14/363/prepub
